# Scaling Up Antiretroviral Treatment Services in Karnataka, India: Impact on CD4 Counts of HIV-Infected People

**DOI:** 10.1371/journal.pone.0072188

**Published:** 2013-08-08

**Authors:** Suresh Shastri, Pavithra Hatna Boregowda, Bharat B. Rewari, Sukarma Tanwar, Anita Shet, Ajay M. V. Kumar

**Affiliations:** 1 Karnataka State AIDS Prevention Society, Bangalore, India; 2 Centre of Excellence, Indira Gandhi Institute of Child Health, Bangalore, India; 3 National AIDS Control Organization, Ministry of Health and Family Welfare, Government of India, New Delhi, India; 4 St. John’s Medical College, Bangalore, India; 5 International Union Against Tuberculosis and Lung Disease, South-East Asia Regional Office, New Delhi, India; McGill University, Canada

## Abstract

**Setting:**

Twelve antiretroviral treatment centres under National AIDS Control Programme (NACP), Karnataka State, India.

**Objective:**

For the period 2004-2011, to describe the trends in the numbers of people living with HIV (PLHIV) registered for care and their median baseline CD4 counts, disaggregated by age and sex.

**Design:**

Descriptive study involving analysis of routinely captured data (year of registration, age, sex, baseline CD4 count) under NACP.

**Results:**

34,882 (97% of total eligible) PLHIV were included in analysis. The number registered for care has increased by over 12 times during 2004-11; with increasing numbers among females. The median baseline CD4 cell count rose from 125 in 2004 to 235 in 2011 – the increase was greater among females as compared to males. However, about two-thirds still presented at CD4 cell counts less than 350.

**Conclusion:**

We found an increasing trend of median CD4 counts among PLHIV presenting to ART centres in Karnataka, an indicator of enhanced and early access to HIV care. Equal proportion of females and higher baseline CD4 counts among them allays any fear of differential access by gender. Despite this relative success, a substantial proportion still presented at low CD4 cell counts indicating possibly delayed HIV diagnosis and delayed linkage to HIV care. Universal HIV testing at health care facilities and strengthening early access to care are required to bridge the gap.

## Introduction

With an estimated 2.5 million people living with HIV (PLHIV), India has the third highest HIV burden in the world, after South Africa and Nigeria [1]. The introduction of antiretroviral therapy (ART) to people living with HIV⁄AIDS has been credited with significantly improving quality of life and reducing mortality [2]. However, a large proportion (15-43%) of HIV-infected individuals in developing countries present themselves for care when CD4 lymphocyte count has fallen below ≤ 200 cells/mm^3^ [1,3]. In 2005, up to 44% individuals presenting at the ART centres of India had baseline CD4 count <100 cells/mm^3^ [4]. Furthermore, till March 2008, 85% had registered for HIV care with baseline CD4 cell count less than 200 cells/mm^3^, indicating advanced stages of immunosuppression due to delays in diagnosis and access to care [5]. Pre-treatment CD4 cell count is one of the important criteria for categorizing the degree of immunosuppression in order to determine eligibility for initiation of antiretroviral therapy. It is well documented that survival rates are longer if ART is started as soon as possible rather than waiting till CD4 counts reach a nadir of <250 cells/mm^3^ [4,6–8]. The consequences of presenting with a low CD4 cell count are multiple; patients are more likely to be diagnosed with severe opportunistic infections, the risk of death may be higher [9], the rate of immunological improvement may be slower [10], the likelihood of transmitting the virus to other individuals is higher, and overall probability of posing a higher financial strain on national health services [11].

**Figure 1:**
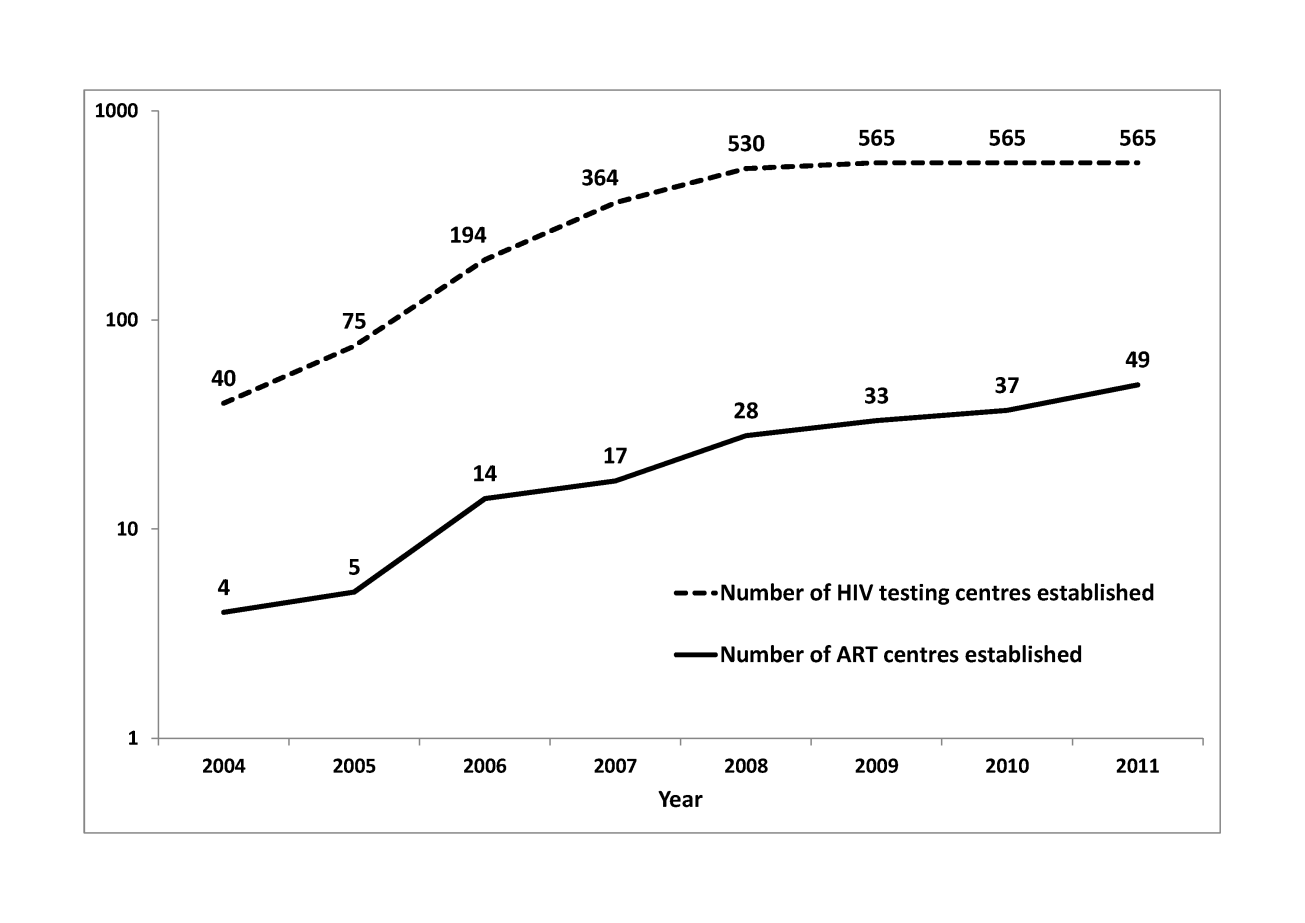
Trends in number of HIV testing centres and ART centres established, year-wise in Karnataka, India, 2004-2011. HIV-Human immunodeficiency virus; ART-antiretroviral therapy. *The Y-axis is in the logarithmic scale.

**Figure 2:**
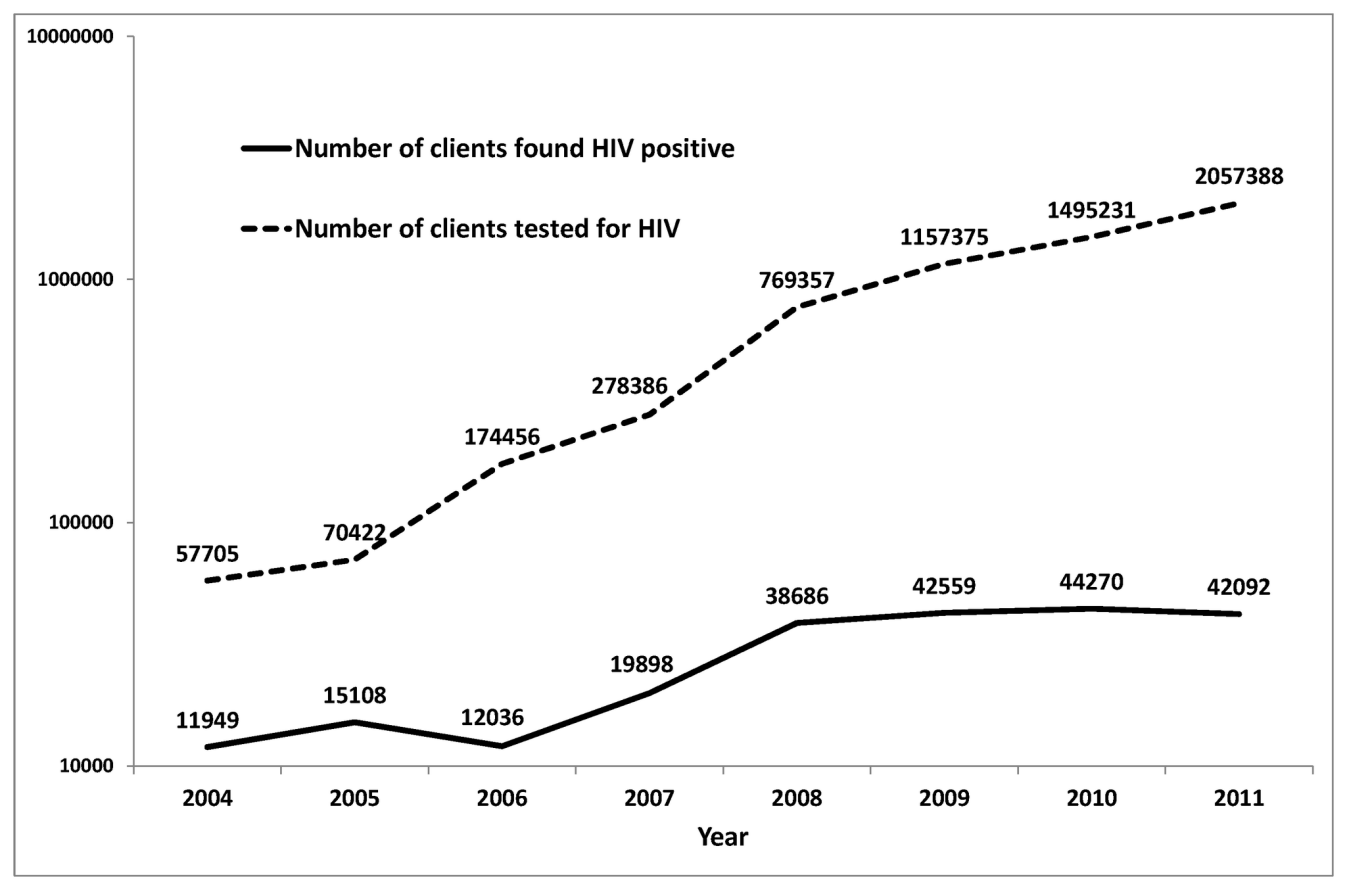
Trends in the number of people tested for HIV and diagnosed HIV positive year-wise, in Karnataka, India, 2004-2011. HIV-Human immunodeficiency virus; ART-antiretroviral therapy. *The Y-axis is in the logarithmic scale.

Karnataka is one of six states in India considered to have high HIV prevalence with an estimated 0.25 million people living with HIV [12]. There has been a massive scale-up of HIV testing and treatment services since 2004. The number of stand-alone HIV testing centres increased from 40 in 2004 to 565 in 2011(Figure 1). This, along with policies to routinely offer HIV testing to TB patients and pregnant women in addition to other high-risk groups were introduced in 2007 which led to an exponential increase in the number of people tested for HIV and found HIV positive (Figure 2). The free ART program for HIV-infected people was launched in Karnataka in four centres in 2004. At present, the state has 49 ART centres with at-least one centre in every district providing multidisciplinary care, counseling and dispensing of ART medications (Figure 1). We hypothesize that this scale-up would have led to early access to care for PLHIV reflected by an increasing trend in average CD4 counts at the time of registration. However, this has not been studied systematically till date. Hence, the specific objectives of this study were:

For the period 2004-2011, in selected 12 ART centres of Karnataka State:

1 To describe the trends in the numbers of PLHIV registered for HIV care, disaggregated by age and sex
2 To describe the trends in median CD4 counts of PLHIV at the time of registration for HIV care, disaggregated by age and sex

## Methods

### Ethics considerations

Since this study was a review of the routinely recorded data and did not involve patient interaction, informed consent was deemed unnecessary. The entire protocol was reviewed for ethical issues by the Ethics Advisory Group of International Union Against Tuberculosis and Lung Diseases (The Union), Paris, France and approved including a waiver of informed consent. Appropriate administrative approvals were obtained by National AIDS Control Organization, New Delhi, India.

### Study Design

This is a descriptive study involving secondary analysis of data routinely recorded under the National AIDS Control Programme (NACP).

### Setting

Karnataka, with 30 districts and a population of 61 million, is one of four large states in South India facing a relatively advanced HIV epidemic, with the adult HIV prevalence in some districts exceeding 1%. As per the NACO report of 2009, Karnataka had a prevalence of 0.63% amounting to 0.25 million persons living with HIV [12].

There are 565 stand-alone HIV testing facilities, 1050 facility integrated HIV testing facilities and 49 ART centres in of the State. Nearly 27 new ART centres in district and sub-district level hospitals were established in the past 4 years. The primary aim of the HIV testing facilities is to provide information, counseling and HIV testing services. All HIV positive persons diagnosed at testing centres are referred to the nearest ART centre for further management. HIV positive patients, who reach ART centres are registered for HIV care, are assessed clinically including CD4 count assessments and if found eligible for ART initiation as per the national guidelines, are initiated on ART [13]. India currently follows WHO 2010 ART guidelines [14]. Since 2008, in its plan to decentralize the monitoring of services, Government of India has established District AIDS Prevention and Control units (DAPCU) headed by a district level officer with support staff for supervision and monitoring in selected districts with high prevalence.

### Study site, Study population, Study period

Twelve ART centres representative of the three geographical zones of the state: north, south and coastal regions of Karnataka were purposively selected for the study based on completeness of data on baseline CD4 count. All PLHIV aged 15 years and above, newly diagnosed and registered to receive HIV care and treatment at each of the selected centres in the state of Karnataka, between April 2004 and December 2011 constituted the study population.

### Data variables and Source

The data were extracted out of the electronic databases maintained in the ART centres during the month of November 2012. Original data sources included the pre-ART patient register and patient treatment cards kept at each centre. The key variables included pre-ART number, year of registration, age in completed years, sex and CD4 lymphocyte count at the time of registration.

### Data management and analysis

Since the data were already present in the electronic format, double data entry and validation was not considered. Abstracted data from the Microsoft Excel database were imported into EpiData [15] software and analyzed. The following key indicators were calculated:

1 Trends in numbers of PLHIV registered for care, year-wise
2 Trends in numbers (proportion) of PLHIV by sex and age groups, year-wise
3 Trends in median (interquartile range) CD4 lymphocyte counts of PLHIV, disaggregated by sex and age groups, year-wise

## Results

Between April 2004 and December 2011, 38,245 newly diagnosed HIV-infected individuals were registered in 12 selected ART centres in north, south, and coastal Karnataka. Of these, 2,367 (6%) were aged <15 years and excluded as per the study criteria. Of the remaining, 34,882 (97%) were included in the final analysis after excluding records with missing information on age, sex and CD4 count.

The key characteristics of the study population and their trends from 2004–11 are shown in Table 1. The number of HIV-infected individuals registered for care has increased by over 12 times from 2004 to 2011. The proportion of females accessing care has gradually increased over the years and is about 52% in 2011 (Chi-square for linear trend; p <0.001). The proportion in the age-group 45 years and above has increased from 11% in 2004 to about 21% in 2011. The median age at the time of registration has increased from 33 years in 2004 to 35 years in 2011 (not shown in the table). The proportion with CD4 count of more than 350 has increased from about 2% in 2004 to over 30% in 2011 (Chi-square for linear trend; p <0.001).

**Table 1:** Trends in characteristics of newly diagnosed HIV-infected individuals registered for HIV care at antiretroviral therapy centres in Karnataka, India, 2004-2011.

**Characteristic**	**2004**	**2005**	**2006**	**2007**	**2008**	**2009**	**2010**	**2011**
**Total number registered**	760	1070	1598	2050	4966	7473	7999	8966
**Sex**								
Male	499 (65.7)	731 (68.3)	950 (59.4)	1180 (57.6)	2601 (52.4)	3905 (52.3)	3967 (49.6)	4335 (48.3)
Female	261 (34.3)	339 (31.7)	648 (40.6)	870 (42.4)	2365 (47.6)	3568 (47.7)	4032 (50.4)	4631 (51.7)
**Age groups (years)**								
15-24	68 (8.9)	84 (7.9)	92 (5.8)	169 (8.2)	463 (9.3)	675 (9.0)	724 (9.1)	869 (9.7)
25-34	349 (45.9)	457 (42.7)	700 (43.8)	880 (42.9)	1896 (38.2)	2870 (38.4)	2895 (36.2)	3272 (36.5)
35-44	259 (34.1)	396 (37.0)	578 (36.2)	713 (34.8)	1756 (35.4)	2547 (34.1)	2737 (34.2)	2996 (33.4)
45-54	60 (7.9)	100 (9.3)	167 (10.5)	213 (10.4)	640 (12.9)	1006 (13.5)	1184 (14.8)	1294 (14.4)
55-64	19 (2.5)	25 (2.3)	50 (3.1)	64 (3.1)	168 (3.4)	298 (4.0)	367 (4.6)	405 (4.5)
> 65	5 (0.7)	8 (0.7)	11 (0.7)	11 (0.5)	43 (0.9)	77 (1.0)	92 (1.2)	130 (1.4)
**Baseline CD4 count (cells/mm^3^)**								
< 50	133 (17.5)	180 (16.8)	410 (25.7)	423 (20.6)	554 (11.2)	775 (10.4)	830 (10.4)	725 (8.1)
51-200	533 (70.1)	726 (67.9)	896 (56.1)	1078 (52.6)	1820 (36.6)	2715 (36.3)	2960 (37.0)	3147 (35.1)
201-350	78 (10.3)	130 (12.1)	171 (10.7)	352 (17.2)	1209 (24.3)	1756 (23.5)	1891 (23.6)	2178 (24.3)
351-500	6 (0.8)	23 (2.1)	78 (4.9)	127 (6.2)	658 (13.3)	1013 (13.6)	1085 (13.6)	1287 (14.4)
501 and above	10 (1.3)	11 (1.0)	43 (2.7)	70 (3.4)	725 (14.6)	1214 (16.2)	1233 (15.4)	1629 (18.2)

Number in parentheses refers to column percentages; HIV = Human ImmunoDeficiency Virus

**Figure 3:**
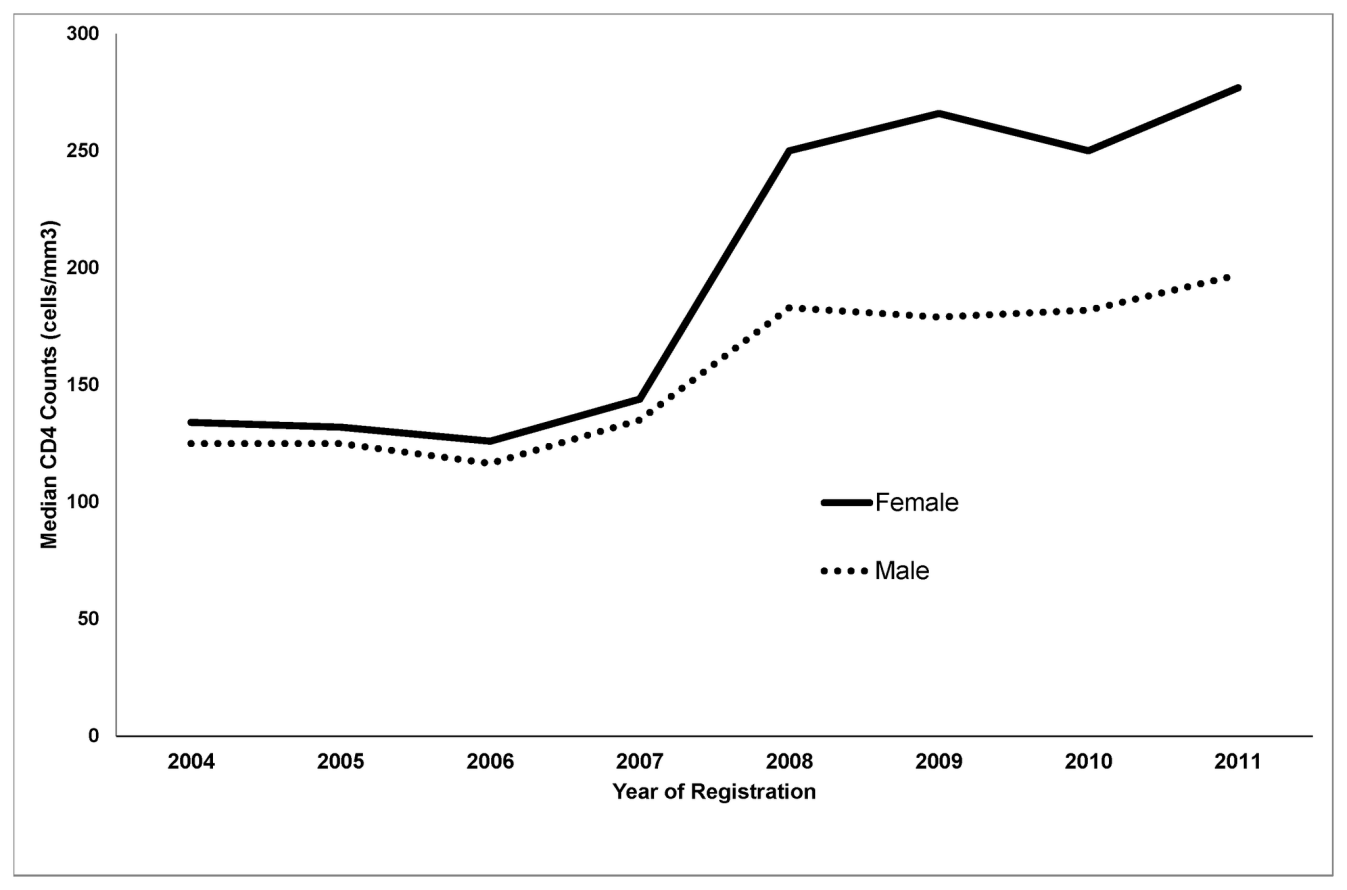
Trends in median baseline CD4 counts of newly diagnosed HIV-infected individuals registered for HIV care at antiretroviral therapy centres, by sex, in Karnataka, India, 2004-2011. HIV-Human immunodeficiency virus; ART-antiretroviral therapy.

Table 2 describes the trends in median CD4 cell counts of HIV-infected individuals from 2004 to 2011. The median CD4 cell count at first presentation for HIV care showed an increasing trend from 125 in 2004 to 235 in 2011. The increase in median CD4 counts was greater among females (Figure 3) and among younger age-groups.

**Table 2:** Trends in median (interquartile range) baseline CD4 counts of newly diagnosed HIV-infected individuals registered for HIV care at antiretroviral therapy centres in Karnataka, India, 2004-2011.

**Characteristic**	**2004**	**2005**	**2006**	**2007**	**2008**	**2009**	**2010**	**2011**
**Total**	125 (69-162)	126 (79-157)	122 (48-165)	136 (62-210)	211 (102-378)	218 (105-397)	213 (106-389)	235 (118-420)
**Sex**								
Male	125 (68-155)	125 (77-156)	117 (47-163)	135 (55-205)	183 (87-318)	179 (88-328)	182 (89-326)	197 (100-358)
Female	134 (74-168)	132 (89-164)	126 (52-169)	144 (69-221)	250 (122-444)	266 (132-466)	250 (129-444)	277 (145-480)
**Age groups (years)**								
15-24	126 (70-165)	145 (102-265)	130 (45-204)	144 (66-217)	365 (190-555)	413 (238-632)	363 (198-549)	405 (248-613)
25-34	124 (65-158)	123 (79-155)	122 (54-165)	137 (56-223)	232 (109-407)	251 (122-438)	246 (130-438)	263 (138-455)
35-44	125 (69-163)	126 (65-163)	120 (45-165)	136 (65-205)	188 (91-320)	179 (89-322)	181 (89-323)	203 (104-362)
45-54	133 (88-153)	125 (92-148)	122 (44-177)	125 (57-199)	172 (81-303)	164 (83-304)	174 (87-318)	183 (86-343)
55-64	132 (95-184)	123 (121-145)	120 (48-163)	134 (62-191)	172 (81-320)	178 (87-290)	156 (77-266)	183 (97-340)
> 65	65 (47-165)	151 (136-165)	96 (49-196)	145 (91-315)	195 (83-253)	144 (83-267)	151 (89-281)	196 (103-311)

Number in parenthesis is IQR = Inter Quartile Range indicating the range from 25^th^ percentile to 75^th^ percentile; HIV = Human ImmunoDeficiency Virus

## Discussion

This is the first study from India systematically examining the scale-up of anti-retroviral treatment services and its potential impact on the trends in average CD4 lymphocyte counts of people living with HIV. The strength of the study was that we collected data from 12 ART centres spread across the state of Karnataka and included all the PLHIV registered for HIV care. We found that there has been a massive scale-up of ART services in the state over the past 8 years with a 12-fold increase in the number of PLHIV registered for care in 2011 as compared to 2004. The proportion of females living with HIV registered for care has consistently increased over the years to reach more than 50% in 2011 allaying any concerns of gender-based inequity in accessing services. Increasing trend in median age of PLHIV registered for care indicates a right shift in age distribution and may be an early indicator of declining HIV epidemic in the state. This fact is confirmed by recent estimates from NACO which indicate a nationally declining trend in HIV incidence and the number of people living with HIV. HIV incidence is declining in both males and females [16]. The rising number of females accessing HIV care in the background of falling incidence provides additional evidence of improved access among females.

It was encouraging to note the increasing trend of median CD4 cell counts from 125 in 2004 to 235 in 2011 indicating improvements in early access to HIV care services. There was a sudden jump in median CD4 count noted from 2007 to 2008 and this could be attributed to several factors that happened in 2007 including introduction of provider initiated HIV testing for TB patients and pregnant women resulting in increase in the number of clients tested for HIV by four times, doubling of HIV positive clients diagnosed and almost doubling of HIV testing centres and ART centres established (Figures 1 and 2). The increase in median CD4 counts was greater among females and among younger age-groups; again a very significant finding indicating improved and early access to HIV care among females. The other possible reasons for the improved access include – widespread advocacy, communication and social mobilization programs, increased support from community programs for HIV counseling and testing and strengthening linkages between patients and care systems.

Despite the increasing trends, more than sixty-five percent of patients continue to first present for HIV care with a CD4 cell count below 350 cells/mm^3^, the level at which initiation of antiretroviral therapy is recommended by national guidelines. The high proportion of patients presenting with low CD4 cell count at their initial clinic visit indicates delayed diagnosis which can lead to high morbidity and mortality [4], higher transmissibility at the community level [17] and steeper treatment costs [18–20].

Few Indian studies in the past have analyzed CD4 trends on such a large state-wide scale. Published national program data of 972 patients at three government ART centres from 2004 to 2005 showed nearly 75% of patients had CD4 cell count <200 cells/mm^3^ at the time of initiation of ART [4]. In New Delhi, India from 2001 to 2007, 33% (n=3680) of patients first presented at CD4 cell count below 200 cells/mm^3^ with 9.5% subjects having CD4 cell count below 50 cells/uL [21]. According to published national program data reporting baseline CD4 cell count of 116,225 registered HIV-infected persons from 2005 to 2008, 85% registered for ART with baseline CD4 cell count less than 200 cells/mm^3^ [5].

Studies have found that male gender and older age are significant determinants of presenting late [22–24]. These have been confirmed in our study as well with a higher median CD4 cell count among females as compared to males. The proportion of females accessing care increased from 2004 to 2011, particularly in 2008 and then after till 2011. The reason for females presenting to care earlier over time may be explained by the fact that females may be getting tested for HIV earlier now through expanded HIV testing programs in pregnancy, or through expanded partner testing programs after a spouse tests positive. Similarly the median CD4 counts among younger age groups were greater when compared to that among older age groups. This can be explained due to the fact that older individuals were likely to have got infected at younger ages, but had a delayed diagnosis [25]. Another study which found similar results explained that younger persons were likely to have been more recently infected compared to older persons, and hence less likely to progress rapidly to develop severe immunosuppression [26].

Our findings have several important programmatic implications for the country. First, increasing average CD4 counts with decentralized access to HIV care is very encouraging and needs to be continued and strengthened. Second, as men are at increased risk of presenting late; further efforts to enroll men into care must be focused. Many untested persons have a perception that they may be at low risk of HIV infection or they are fearful of being aware of their HIV status [27]. Such persons are more likely to present at late stage or have an illness-triggered HIV diagnosis. Our findings reinforce the need to establish universal routine HIV testing as standard of care for all adolescents and adults seen in private and public care settings, regardless of patient reported HIV risk [28]. It is only under such circumstances that late-stage or illness-triggered HIV diagnoses will be reduced. Third, this study provides valuable information useful for program planning. The information on proportions of PLHIV in several CD4 groups helps the programme manager to assess the possible increase in workload at ART centres with changes in ART initiation criteria from a CD4 threshold of 200 to 350, or from 350 to 500. In light of the recently released ART guidelines by the WHO [29,30], this information helps in planning for drug procurement and distribution of antiretroviral drugs.

As with any operational research, there were a few limitations. While we selected 12 ART centres representing the three regions of Karnataka and have no reason to believe that they are any different from the rest of the ART centres in the state, we have no data to demonstrate the same. Similarly, we had to exclude children from the study owing to incomplete data among them. We also acknowledge the limitations of using ecologic data in measuring access to HIV services.

In conclusion, we have found that in Karnataka, there has been a massive scale-up of HIV diagnostic and treatment services and it has improved the median CD4 cell counts of PLHIV at the time of registration, an indicator of early access to care. However, about two-thirds were diagnosed with CD4 cell counts ≤ 350cells/ mm^3^, the threshold at which initiating ART is unequivocally recommended as per national guidelines [13,14]. These findings suggest that further expanding HIV testing and reducing late HIV diagnosis needs to be a priority, if the programs related to improving linkage to care and earlier antiretroviral treatment initiation are to reach patients and potentially alter the trajectory of the HIV epidemic in India.
